# Serum osteocalcin level is associated with the mortality in Chinese patients with Fibrodysplasia ossificans progressiva aged ≤18 years at diagnosis

**DOI:** 10.1186/s12891-020-3170-3

**Published:** 2020-03-06

**Authors:** Dunmin She, Ran Li, Ping Fang, Guannan Zong, Ying Xue, Keqin Zhang

**Affiliations:** Department of Endocrinology and Metabolism, Tongji Hospital of Tongji University, Tongji University School of Medicine, No. 389, Xincun Road, Shanghai, 200065 China

**Keywords:** Fibrodysplasia ossificans progressiva (FOP), Mortality, Risk factors, Osteocalcin

## Abstract

**Background:**

Fibrodysplasia ossificans progressiva (FOP) is an ultra-rare genetic disorder characterized by extraskeletal heterotopic ossification. It is well recognized that FOP can lead to a devastating condition of disability. However, the mortality rate of FOP patients in China and risk factors for mortality are still largely unclear.

**Methods:**

We conducted a retrospective research on a cohort of 65 cases of FOP patients in China from 2008 to 2018. We reviewed medical records of these FOP patients to retrieve information such as date of birth/death, gender, clinical features, genotypes and biochemical parameters and analyze the correlation of these parameters with the mortality.

**Results:**

92.3% (60/65 cases) patients were classic FOP patients, 3.1% (2/65 cases) were FOP-plus and 4.6% (3/65 cases) were FOP variants. 9 cases of this cohort were dead during the ten-year period, and the overall mortality rate was 13.8%. c.617G > A mutation was confirmed in all non-survivors. In FOP patients≤18 years at diagnosis, non-survivors demonstrated significantly lower blood osteocalcin and alkaline phosphatase levels compared with survivors (*P* < 0.05), and spearman correlation and logistic regression analysis indicated that serum osteocalcin and alkaline phosphatase levels were negatively correlated with the mortality. Furthermore, the receiver-operating characteristic curve analysis showed serum osteocalcin had the largest area under the curve of 0.855 among four biochemical parameters, and serum osteocalcin < 65.9 ng/ml displayed a good capacity to discriminate the non-survivors from survivors in FOP patients aged 18 years and younger at diagnosis.

**Conclusions:**

Our findings showed that the mortality rate of FOP was 13.8% in China. Serum OC level was negatively correlated with the mortality in Chinese FOP patients ≤18 years at diagnosis.

## Introduction

Fibrodysplasia ossificans progressiva (FOP) (Mendelian Inheritance in Man [MIM] #135100), also known as myositis ossificans progressiva, is a rare genetic disease characterized by heterotopic ossification (HO) in anatomical sites and congenital skeletal malformations [1, 2] which eventually cause disability. Most patients have congenital malformation of great toes at birth and develop painful inflammatory soft tissue swelling (flare-up) during the first decade of life spontaneously or by trauma [1]. Accumulative painful swelling gradually turns into multiple, and eventually causes joint and bone deformities as well as other disabilities [1]. FOP is an extremely rare disease with prevalence of 1 case in 2 millions in the world [3]. For this reason, patients with FOP are at a high risk of being misdiagnosed and/or under-diagnosed [4, 5]. FOP is primarily caused by de novo mutation in the Glycine Serine (GS) activation domain of type I activin A receptor (ACVR1), a bone morphogenetic protein (BMP) type I receptor [6, 7]. Approximately 97% of FOP individuals have ACVR1 (c.617G > A; p.R206H) mutation [6]. Presently, clinical managements for FOP are palliative and symptomatic, which is insufficient to completely prevent long-term complications [8].

Ankylosis of affected joint, which is the most common complication, may result in progressive immobility and lifelong wheelchair-bound [9]. Submandibular swelling can be a life-threatening complication, especially when the patient has difficulty in swallowing [9]. Thoracic insufficiency syndrome usually causes cardiorespiratory failure and pneumonia, which have been reported to be the two most common causes of death in FOP patients [10]. Although much is known about the lethal complications of FOP, few researches reported the mortality of FOP worldwide. By far, there has been only one previous research which described the mortality rate of FOP patients for the first time in the world [10].

It is estimated that there are at least 650 cases of FOP patients in China, the most populous country in the world [11]. However, only a small fraction of Chinese patients with FOP have been reported. Previously, we have reviewed the epidemiology and the research progress of FOP in China [12]. Here, in order to analyze the mortality rate and the risk factors for the mortality, we conducted a retrospective study on a cohort of 65 cases with FOP from 2008 to 2018, and analyzed the medical history, clinical features, genotypes and biochemical parameters of those patients.

## Methods

### Patients

From December 2008 to December 2018, 65 cases of individuals who were diagnosed as FOP in Tongji Hospital of Tongji University were recruited, consisting of 30 males and 35 females. The diagnosis of FOP was based on the two clinical features [3]: 1) characteristic congenital malformation of the great toes at birth and 2) progressive extraskeletal endochondral ossification. According to clinical classification schemes for FOP [3], 65 individuals were categorized as 60 cases of classic FOP based on classic defining features of FOP, 2 cases of FOP-plus according to atypical features such as cryptorchidism or childhood glaucomain addition to the classic defining features, and 3 patients of FOP variants for their mild malformations of great toes. The diagnosis of FOP in those 65 patients was confirmed based on the clinical manifestations by at least two investigators combined with ACVR1 gene analysis. Informed consent was obtained from all participants. Our study was approved by the investigational review board of Tongji Hospital of Tongji University.

### Methods

The medical records were reviewed and the following data were collected in FOP patients: date of birth/death, birthplace, gender, personal history, family history, clinical manifestations, physical examinations, and blood biochemical tests. All blood tests were performed at the clinical laboratory of Tongji Hospital of Tongji University. Blood biochemical tests included serum parathyroid hormone level (PTH reference range, 15–65 pg/mL, Roche kit), osteocalcin (OC,10–70 ng/mL, Roche kit), total serum calcium (Ca, 2.2–2.65 mmol/L), alkaline phosphatase (ALP 50–130 U/L), phosphorus (P, 0.81–1.45 mmol/L), erythrocyte sedimentation rate (ESR, 0-20 mm/h), alanine aminotransferase (ALT, 9-50 U/L), aspartate aminotransferase (AST, 15-40 U/L), creatinine (Cr, 44–133 umol/L), and blood uric nitrogen (BUN, 2.9–8.2 mmol/L). According to the previous protocols [3], genomic DNA from blood samples of 50 patients were genotyped for mutations in ACVR1 when their biochemical tests were performed simultaneously. The rest 15 cases were clinically diagnosed without gene analysis. We determined the lifespan by telephone follow-up or outpatient interview in the second half of 2018. All FOP patients were divided into four subgroups according to their prognosis and their ages at diagnosis: non-survivors ≤18 years at diagnosis (group A), survivors ≤18 years at diagnosis (group B), non-survivors aged >18 years at diagnosis (group C) and survivors aged >18 years at diagnosis (group D). All blood tests were performed when they first admitted to our center (which was also the time when they were firstly diagnosed as FOP). None of the FOP patients had taken any medications including glucocorticoid within 1 month before admission to our center.

### Statistical analysis

Statistical package for social sciences (SPSS) 19.0 was used for the statistical analysis. The Kolmogorov-Smirnov test was used to determine whether the parameters followed a normal distribution. All continuous variables were presented as mean ± standard deviation. Data were illustrated as medians (interquartile ranges) if not conforming to normal distribution. Comparisons of the means and proportions were performed with independent samples t-test and the chi-square test, respectively. Spearman correlations and logistic regression analysis were performed to explore the risk factors for the mortality of FOP patients. Receiver-operating characteristic (ROC) curves were constructed to estimate the cut-off, sensitivity and specificity of the risk factors which potentially predicted the early death of FOP patients. Tests were two-sided and a *P*-value < 0.05 was considered significant.

## Results

### Clinical features of FOP patients

65 cases of FOP individuals recruited in our study were all Han people from 25 provinces of China. 46.2% (30/65 cases) were male while 53.8% (35/65 cases) were female. According to criterion of classification, 92.3% (60/65 cases) patients were diagnosed as classic FOP patients based on classic defining features of FOP, 3.1% (2/65 cases) of individuals were FOP-plus since they had classic defining features of FOP and other atypical features such as cryptorchidism or childhood glaucoma, and 4.6% (3/65 cases) were determined as FOP variants for their mild malformations of great toes. ACVR1 gene analysis was performed in 50 patients and revealed that 48 cases were canonical c.617G > A mutation. Two of FOP variants did not present canonical c.617G > A mutation: one of FOP variants showed a variant mutation in ACVR1 at c.774G > C (p.R258S), while the rest one had a mutation in ACVR1 at c.1067G > A (p.G356D). (Table 1) In the 65 cases of FOP patients, 84.6% (55 cases) of FOP patients were initially misdiagnosed before their first visit to our clinic, and approximately 36.9% of patients (24 cases) had undergone unnecessary biopsies.

**Table 1:** Clinical Features of FOP patients

	Classic FOP	FOP-plus		FOP variants		
**Patient number**	60cases	46	70	7	42	54
**ACVR1 mutation**						
-Coden change	R206H (45 cases) or not complete (15 cases)	R206H	R206H	R258S	R206H	C306D
-Nucleotide change (cDNA position)	c.617G > A (45 cases) or not complete(15 cases)	c.617G > A	c.617G > A	c.744G > C	c.617G > A	c.1067G > A
**Gender**	M&F (26/34)	M	M	M	M	F
**Age of onset (years)**	0–13	18	5	6	2	2.5
**Typical FOP features**						
-Congenital malformations of Great toes	Y	Y	Y	N	Y	Y
-progressive HO	Y	Y	Y	Y	Y	Y
-onset site	Scalp(45%); Neck(20%); Back(15%); Shoulder(6.7%); Hip (5%); Others (8.3%)	Shoulder	Back	Knee	Scalp	Shoulder
**Other typical FOP features**						
-Thumb malformations	1.7%	Y	Y	Y	Y	N
-Knee osteochondromas	51.7%	N	N	Y	N	N
- Scoliosis	56.7%	N	N	Y	N	Y
-Conductive hearing impairment	5%	N	N	N	N	N
**Atypical FOP features features**	N	Childhood glaucoma	Cryptorchidism	Minimal changes in great toes	No changes in great toes	Minimal changes in great toes

### Mortality rate of FOP patients

During the past 10-year period (Dec 2008 to Dec 2018), 9 patients with FOP (3 males and 6 females) were dead while 56 patients (27 males and 29 females) were survival. Hence, the total mortality rate was 13.8% (9/65 cases). The mortality rate in FOP patients aged 18 years and younger at diagnosis was 11.1% (5/45 cases), whereas it was 20% (4/20 cases) in patients aged over 18 years. All non-survivors were confirmed c.617G > A mutation, and were diagnosed as classic FOP for their congenital malformations of great toes and progressive HO (Table 2). The average age at diagnosis (age at first admission to our clinic) of 65 cases was 14.4 ± 8.8 years. Among all non-survivors, 5 cases were 18 years and younger at diagnosis, and the rest 4 cases were over 18 years at diagnosis. The average age at diagnosis of non-survivors over 18 years and non-survivors aged 18 years and younger were 25.5 ± 2.4 years and 11.6 ± 6.3 years. The average age of onset (first flare-up or HO) of all FOP patients was 4.2 ± 3.8 years. There was no significant difference in age at onset between non-survivors over 18 years at diagnosis and non-survivors aged 18 years and younger at diagnosis. The average age at death of all non-survivors was 22.8 ± 8.9 years. The age of death in non-survivors over 18 years at diagnosis was significantly older than that in non-survivors aged 18 years and younger at diagnosis (29.5 ± 2.9 years vs17.4 ± 7.2 years) (Table 3).

**Table 2:** Clinical Features of FOP non-survivors

Patient number	1	2	8	21	34	39	41	45	98
**Gender**	F	M	F	M	F	M	F	F	F
**Age of onset**	4	3	2	0.1	6	0.7	2.5	1	6
**Age of diagnose (years)**	>18	>18	≤18	≤18	>18	>18	≤18	≤18	≤18
**Age of death (years)**	>18	>18	>18	≤18	>18	>18	>18	≤18	>18
**Course of disease (years)**	1	6	6	4	6	3	6	6	7
**ACVR1 mutation**									
-Coden change	R206H	R206H	R206H	R206H	R206H	R206H	R206H	R206H	R206H
-Nucleotide change (cDNA position)	c.617G > A	c.617G > A	c.617G > A	c.617G > A	c.617G > A	c.617G > A	c.617G > A	c.617G > A	c.1067G > A
**Typical FOP features**									
-Congenital malformations of Great toes	Y	Y	Y	Y	Y	Y	Y	Y	Y
-progressive HO	Y	Y	Y	Y	Y	Y	Y	Y	Y
-onset site	Scalp	Neck	Neck	Shoulder	Back	Scalp	Scalp	Scalp	elbow
**Classification**	classic FOP	classic FOP	classic FOP	classic FOP	classic FOP	classic FOP	classic FOP	classic FOP	classic FOP
**Biochemical tests**									
-OC (u/L)	38.1	40.7	12.7	64.7	6.58	41.5	43.9	64.8	38.0
-Ca (mmol/L)	2.3	2.35	2.08	2.4	2.24	2.3	2.62	2.24	2.21
-P (mmol/L)	1.16	1.42	1.21	1.45	0.72	1.2	1.34	1.65	1.02
-PTH (pg/ml)	30.9	95.4	90.9	29	53.3	86.5	37.8	50.9	78.7

**Table 3:** The baseline characteristics of survivors and non-survivors

FOP patients	≤18y		>18y		Total
	survivors (group A)	non-survivors (group B)	survivors (group C)	non-survivors (group D)	
Cases	40(88.9%)	5(11.1%)	16(80%)	4(20%)	65
Gender ratio (M/F)	21/19	1/4	6/10	2/2	30/35
Age at diagnose (years)	9.6 ± 5.0	11.6 ± 6.3	24.4 ± 7.1 ^**△△^	25.5 ± 2.4 ^**△△^	14.4 ± 8.8
Age of onset	3.6 ± 2.9	2.3 ± 2.3	4.6 ± 4.4	3.4 ± 2.2	4.2 ± 3.9
Age at death (years)	–	17.4 ± 7.2	–	29.5 ± 2.9^△△^	22.8 ± 8.9
OC (ng/mL)	87.7 ± 32.7	48.7 ± 15.5^**^	34.7 ± 19.8^**^	26.8 ± 16.1^**^	67.9 ± 37.8
Ca (mmol/L)	2.32 ± 0.2	2.34 ± 0.07	2.32 ± 0.08	2.24 ± 0.05	2.32 ± 0.15
P (mmol/L)	1.58 ± 0.25	1.42 ± 0.15	1.23 ± 0.3^**^	1.02 ± 0.21^**△^	1.45 ± 0.31
PTH (pg/mL)	63.3 ± 487.4	53.2 ± 25.9	81.5 ± 37.7	71.9 ± 14.3	67.5 ± 42.8
ALP (IU/L)	244.0 ± 121.1	128.2 ± 85.0^*^	121.9 ± 78.6^**^	82.2 ± 24.9^*^	192.3 ± 121.9
ESR (mm/hr)	7.9 ± 5.2	9.4 ± 8.7	10.2 ± 9.2	9.25 ± 5.6	8.6 ± 7.8
ALT (U/L)	14.9 ± 7.2	10.6 ± 3.4	15.0 ± 10.6	16.7 ± 13.2	14.6 ± 8.0
AST (U/L)	24.6 ± 9.7	23.1 ± 6.0	25.3 ± 10.9	19.57 ± 12.7	24.3 ± 13.3
Cr (umol/L)	38.0 ± 14.3	31.0 ± 5.2	46.8 ± 14.0	43.7 ± 11.3	39.4 ± 14.0
BUN (mmol/L)	2.0–5.8	1.7–7.2	2.9–6.3	2.2–5.3	1.7–7.2

Compared with group A, ^*^P < 0.05, ^**^P < 0.01; Compared with group B, ^△^*P* < 0.05, ^△△^*P* < 0.01;

*OC* osteocalcin, *Ca* total serum calcium, *P* phosphorus, *PTH* parathyroid hormone, *ALP* alkaline phosphatase, *ESR* erythrocyte sedimentation rate, *CRP* C-reactive protein, *ALT* alanine aminotransferase, *AST* aspartate aminotransferase, *Cr* creatinine, *BUN* blood urea nitrogen

### Risk factors for the mortality of FOP patients

The clinical and biochemical data of the survival and non-survival FOP patients were shown in Table 3. In FOP patients aged 18 years and younger at diagnosis, serum OC and ALP levels were significantly lower in non-survival patients compared with survivors. Besides, there was no significant difference in gender ratio, age at diagnosis, serum Ca, P, PTH, ESR, ALT, AST, Cr or BUN at first visit between those two groups. In FOP patients aged over 18 years at diagnosis, there was no significant difference in gender ratio, age at diagnosis, serum OC, Ca, P, ALP, PTH, ESR, ALT, AST, Cr or BUN between survivors and non-survivors (Table 3).

Spearman correlation analysis showed that serum OC, P, ALP were negatively correlated with the mortality in the total FOP patients (supplementary Table 1), whereas serum OC, ALP were negatively associated with the mortality in FOP patients ≤18 years at diagnosis (supplementary Table 2). No correlation between the mortality and the blood biochemical parameters was found in FOP patients >18 years at diagnosis (supplementary Table 3).

Logistic regression analysis indicated that serum OC level was negatively associated with the mortality in all FOP patients and FOP patients aged 18 years and younger at diagnosis. Further adjustment for age and gender did not change the correlation between serum OC and the mortality in all FOP patients (OR: -1.043; *P* = 0.030) and FOP patients ≤18 years at diagnosis (OR: -1.224; *P* = 0.028). However, in FOP patients aged over 18 years at diagnosis, no association between serum OC level and the mortality was found.

**Fig. 1:**
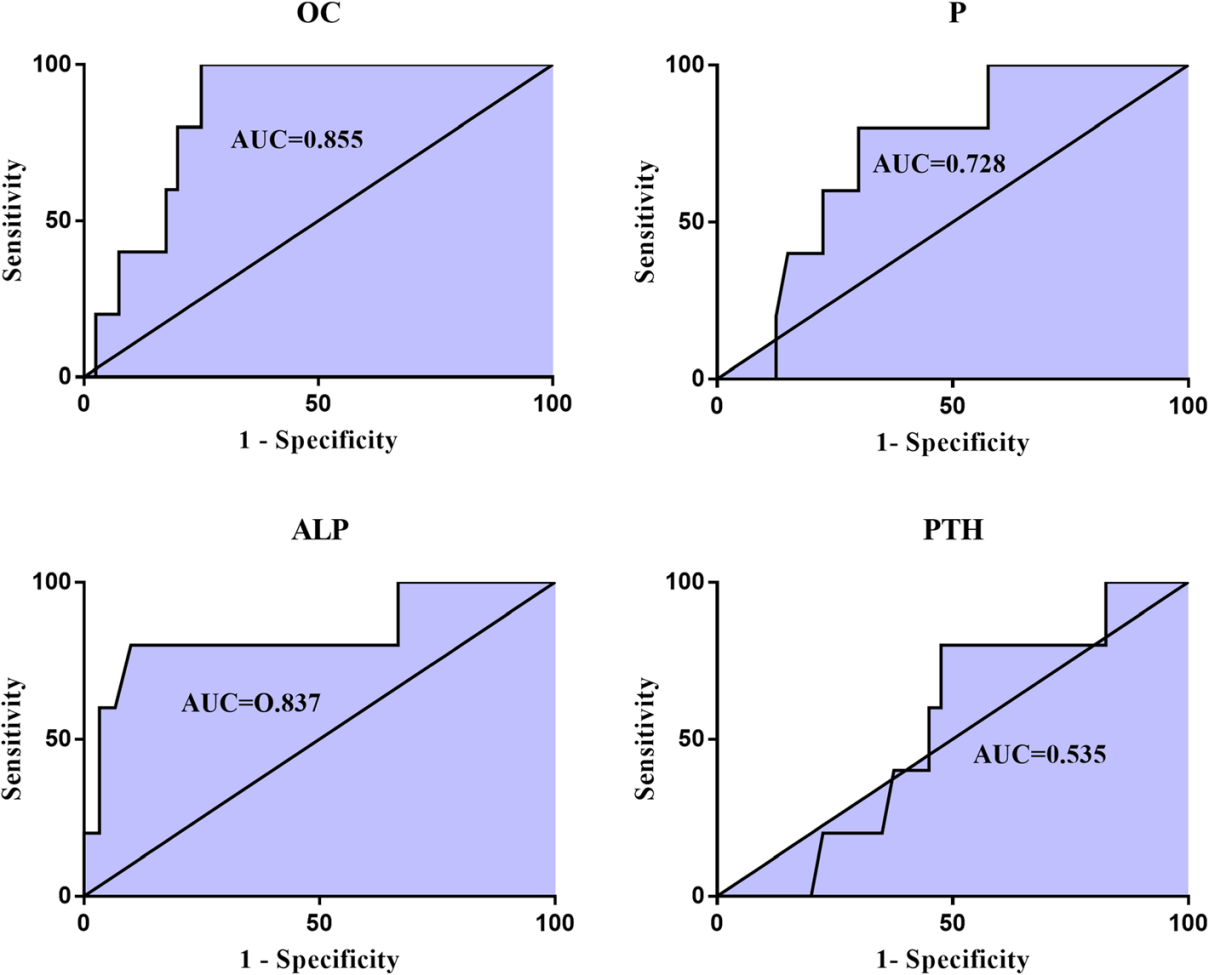
Receiver-operating characteristic (ROC) curves for the ability of serum OC, P, ALP and PTH to discriminating the non-survivors from the survivors in FOP patients with age ≤ 18y. CI, confidence interval. The area under the ROC curve (AUC) = 0.855 for OC, *P* = 0.010, 95%CI 0.739–0.971; AUC = 0.728 for P, *P* = 0.100, 95%CI 0.547–0.908; AUC = 0.837 for ALP, *P* = 0.017, 95%CI 0.605–1.068; AUC = 0.535 for PTH, *P* = 0.800, 95%CI 0.320–0.750. OC: osteocalcin; P: phosphorus; ALP: alkaline phosphatase; PTH: parathyroid hormone

ROC curves were constructed to evaluate the sensitivity and specificity of the blood biochemical parameters as potential factors to discriminate the non-survivors from survivors of FOP patients aged 18 years and younger at diagnosis. The areas under the ROC curves (AUCs) of blood biochemical markers were shown in Fig. 1. Among four blood biochemical markers, serum OC had the largest AUC of 0.855, and serum OC < 65.9 ng/mL displayed a sensitivity of 100% and specificity of 75% in discriminating the non-survival patients from the survivors in FOP patients ≤18 years at diagnosis. Furthermore, the AUC of serum ALP, P and PTH was 0.837, 0.728 and 0.535 separately. The cut-off points of those four blood biochemical markers as predictors for the mortality in FOP patients aged 18 years and younger at diagnosis were presented in Table 4.

**Table 4:** Area under the Curve and related parameters

	AUC	Cut-off point	Sensitivity	Specificity	*P*-value	95%CI
OC	0.855	< 65.9 ng/mL	100%	75%	0.010^*^	0.739–0.971
P	0.728	< 1.47 mmol/L	80%	70%	0.100	0.547–0.908
ALP	0.837	< 122.9 IU/L	80%	90%	0.017^*^	0.605–1.068
PTH	0.535	< 55.7 pg/mL	80%	52.5%	0.800	0.320–0.750

^*^P <0.05

*OC* osteocalcin, *P* phosphorus, *ALP* alkaline phosphatase, *PTH* parathyroid hormone

## Discussion

Previous researches of FOP usually focused on clinical features and genetic patterns of FOP patients [1, 13, 14]. However, few study described clinical outcomes in FOP patients. Until recently, only one study of mortality and median lifespan in FOP patients has been reported. That study retrospectively reviewed mortality records of 144 cases from two large registries of FOP patients worldwide, and found the mortality rate was 13.9% [10]. In our study with Chinese FOP patients, the mortality rate was 13.8%, which was similar to the previous study [10]. However, risk factors for the mortality in FOP patients are still largely unknown.

In our study, we found serum OC level was negatively associated with the mortality in FOP patients, and this correlation only exist in the FOP patients aged 18 years and younger at diagnosis. OC is a major non-collagenous protein in bone and serves as a biomarker of bone formation mostly. Serum level of OC is significantly correlated with the age [15]. In previous study, serum OC level reaches its peak in girls aged 11–14 years and boys aged 13–15 years. Then, it begins to decrease at the age of 20 years in males and females [16]. It has been reported that the serum OC level is 50–150 ng/mL in children, whereas it is 12–20 ng/mL in healthy adults [17]. In our study, the OC level was 87.7 ± 32.7 ng/mL in survivors of FOP patients when diagnosed ≤18 years, which was within the reference range of serum OC level in children according to the previous studies [16, 17]. However, serum OC level in non-survivors of FOP patients ≤18 years at diagnosis (48.7 ± 15.5 ng/mL), which was below the reference range in the previous studies, was significantly lower than that in survivors. Therefore, we speculate that serum OC level might serve as a predictor for the mortality in FOP patients ≤18 years at diagnosis as serum OC levels of non-survivors in this age group were below the reference range. However, our conjecture still needs to be confirmed by follow-up studies in the future.

Cardiorespiratory failure from thoracic insufficiency syndrome was a severe complication and a common cause of death in FOP patients [10]. It has been reported that dysregulations of BMP pathway may make an impact on vascular dysfunctions, while overactive BMP signaling in FOP may play a central role in the development of hypoxia-induced pulmonary hypertension [18]. Recent studies have demonstrated serum OC level is closely associated with cardiovascular disease. Zhang et al. [19] found that lower serum OC levels were associated with a higher risk of major adverse cardiovascular events in a retrospective cohort study of Chinese subjects. A longitudinal analysis demonstrated that men with low OC levels exhibit increased all-cause mortality and cardiovascular disease related mortality [20]. In our study, we found that serum OC level was negatively correlated with the mortality in FOP patients ≤18 years at diagnosis. Therefore, we speculate that serum OC level was negatively associated with the mortality in FOP patients aged 18 years and younger at diagnosis might due to its relationship with dysfunction of cardiopulmonary system. However, the relationship between serum OC level and the cause of death in FOP patients still needs to be clarified in the future study.

Bone transformation markers including OC are highly dependent on gender, age and pubertal stage [21, 22]. In our analysis, after adjusting for age and gender, serum OC level was negatively associated with the mortality in all FOP patients and FOP patients aged 18 years and younger at diagnosis. We did not analyze the effect of pubertal stage on biomarkers due to small size of samples. In addition, since the identification of the severity of FOP is still unclear, we are not sure if the severity of FOP has an effect on serum biomarkers.

Our research has several limitations. Firstly, 65 of FOP patients were recruited in our study, which only represent 10 % of all FOP patients in China [11]. Secondly, it is a retrospective study without long-term follow-up. Serum biochemical parameters of FOP patients may fluctuate dynamically considering the long-lasting process of this disease. Thus, further follow-up study is needed to confirm our result in a larger cohort of patients.

## Conclusions

In summary, our study described the mortality rate and related risk factors for the mortality of FOP patients in China for the first time. We found that the mortality rate of FOP was 13.8% and serum OC level was negatively correlated with the mortality in Chinese FOP patients ≤18 years at diagnosis. Our study could be valuable in predicting lethal outcome of FOP patients aged 18 years and younger at diagnosis, allowing interventions to be conducted most effectively to reduce the associated mortality.

## Supplementary information

**Additional file 1: Supplementary Table 1.** Spearman correlations between parameters and mortality in FOP patients. **Supplementary Table 2.** Spearman correlations between parameters and mortality in FOP patients with age≤18 years. **Supplementary Table 3.** Spearman’s correlations between parameters and mortality in FOP patients with age>18 years.

## Data Availability

The datasets used and analyzed during the current study are available from the corresponding author on reasonable request.

## Abbreviations

ACVR1: Type I activin A receptor
ALP: Alkaline phosphatase
ALT: Alanine aminotransferase
AST: Aspartate aminotransferase
AUCs: Areas under the ROC curves
BMP: Bone morphogenetic protein
BUN: Blood uric nitrogen
Ca: Calcium
Cr: Creatinine
ESR: Erythrocyte sedimentation rate
FOP: Fibrodysplasia ossificans progressiva
GS: Glycine Serine
HO: Heterotopic ossification
OC: Osteocalcin
P: Phosphorus
PTH: Parathyroid hormone
ROC: Receiver-operating characteristic
SPSS: Statistical package for social sciences
