# Supplementary material for: Serum osteocalcin level is associated with the mortality in Chinese patients with Fibrodysplasia ossificans progressiva aged ≤18 years at diagnosis
Source: BMC Musculoskelet Disord. 2020 Mar 6;21:152. doi: 10.1186/s12891-020-3170-3 (PMC7060591; doi:10.1186/s12891-020-3170-3)
Supplement: Supplementary file 1 — Additional file 1: Supplementary Table 1. Spearman correlations between parameters and mortality in FOP patients. Supplementary Table 2. Spearman correlations between parameters and mortality in FOP patients with age≤18 years. Supplementary Table 3. Spearman’s correlations between parameters and mortality in FOP patients with age>18 years. [file 12891_2020_3170_MOESM1_ESM.doc]

**supplementary Table 1 Spearman correlations between parameters and mortality in FOP patients**

| parameter |  | Gender | Age | OC | Ca | P | PTH | ALP | ESR | ALT | AST | BUN | Cr |
| --- | --- | --- | --- | --- | --- | --- | --- | --- | --- | --- | --- | --- | --- |
| Mortailty | R | -0.103 | 0.170 | -0.313 | -0.179 | -0.280 | 0 | -0.332 | -0.039 | -0.210 | -0.095 | -0.064 | -0.200 |
|  | P | 0.414 | 0.176 | 0.011^*^ | 0.153 | 0.024^*^ | 1 | 0.020^*^ | 0.766 | 0.143 | 0.514 | 0.670 | 0.670 |

^*^ *P* < 0.05;

OC: osteocalcin； Ca: total serum calcium; P: phosphorus; PTH: parathyroid hormone; ALP: alkaline phosphatase ; ESR: erythrocyte sedimentation rate; ALT: Alanine aminotransferase; AST: Glutamic oxaloacetic transaminase; BUN: blood urea nitrogen; Cr: creatinine

**supplementary Table 2 Spearman correlations between** parameters and mortality in FOP patients with age≤18 years

| parameter |  | Gender | Age | OC | Ca | P | PTH | ALP | ESR | ALT | AST | BUN | Cr |
| --- | --- | --- | --- | --- | --- | --- | --- | --- | --- | --- | --- | --- | --- |
| Mortality | R | -0.204 | 0.112 | -0.387 | -0.038 | -0.248 | -0.038 | -0.367 | -0.058 | -0.289 | -0.130 | -0.166 | -0.295 |
|  | P | 0.178 | 0.464 | 0.009^**^ | 0.804 | 0.101 | 0.804 | 0.026 | 0.706 | 0.083 | 0.445 | 0.341 | 0.085 |

^*^ *P* < 0.05，^**^ *P* < 0.01;

OC: osteocalcin； Ca: total serum calcium; P: phosphorus; PTH: parathyroid hormone; ALP: alkaline phosphatase ; ESR: erythrocyte sedimentation rate; ALT: Alanine aminotransferase; AST: Glutamic oxaloacetic transaminase; BUN: blood urea nitrogen; Cr: creatinine

**supplementary Table 3 Spearman’s correlations between parameters and mortality in FOP patients with age＞18 years**

| parameter |  | Gender | Age | OC | Ca | P | PTH | ALP | ESR | ALT | AST | BUN | Cr |
| --- | --- | --- | --- | --- | --- | --- | --- | --- | --- | --- | --- | --- | --- |
| Mortality | R | -0.102 | -0.241 | 0.152 | 0.414 | 0.238 | -0.065 | 0.194 | 0.085 | 0.024 | 0.049 | -0.194 | 0.130 |
|  | P | 0.669 | 0.306 | 0.523 | 0.070 | 0.311 | 0.785 | 0.545 | 0.745 | 0.937 | 0.874 | 0.545 | 0.688 |

OC: osteocalcin； Ca: total serum calcium; P: phosphorus; PTH: parathyroid hormone; ALP: alkaline phosphatase ; ESR: erythrocyte sedimentation rate; ALT: Alanine aminotransferase; AST: Glutamic oxaloacetic transaminase; BUN: blood urea nitrogen; Cr: creatinine
